# Primary Pancreatic Hydatid Disease With Secondary Peritoneal Dissemination Presenting as Acute Abdomen Caused by Cyst Rupture: A Report of a Rare Case

**DOI:** 10.7759/cureus.105108

**Published:** 2026-03-12

**Authors:** Farah Al-Shabot, Meharish Ambatt, Omar M Al-Marzouqi

**Affiliations:** 1 Medicine, Dr. Sulaiman Al-Habib Hospital, Dubai, ARE; 2 College of Medicine, Dubai Medical University, Dubai, ARE; 3 General Surgery, Mediclinic Parkview Hospital, Dubai, ARE; 4 General Surgery, Dubai Hospital, Dubai, ARE

**Keywords:** complex hydatid cyst, extra pancreatic complications, multiorgan hydatid disease, multiple hydatid cysts, pancreatic cystic neoplasms, pancreatic hydatid cyst, pancreatic tail hydatid cyst, primary pancreatic hydatid cyst

## Abstract

Hydatid disease is a parasitic infection caused by the cestode Echinococcus granulosus, which most commonly affects the liver due to spread through the portal circulation, but the parasite may also escape into the systemic circulation and involve other organs, such as the pancreas. Pancreatic hydatid cysts can localize in any part of the pancreas, such as the head, body, or tail. Primary localization to the tail of the pancreas with secondary dissemination after an acute cyst rupture can make diagnosis more challenging. Diagnosis is often missed early due to multiple factors, such as low clinical suspicion from an atypical disseminated cyst mass-effect presentation, which can mask pancreatic pathology and possibly mimic urinary pathology, as in our patient. Diagnostic difficulty can also be attributed to the similar appearance that pancreatic hydatid cysts may share with other cystic lesions on imaging. This case report highlights the diagnostic challenges in a rare case of primary pancreatic hydatid disease of the tail with secondary peritoneal dissemination in a 29-year-old African male who presented to the emergency department with generalized abdominal pain and urinary symptoms of dysuria, increased urgency, and frequency. Definitive diagnosis in such cases can be reached with thorough assessment of exposure history and imaging, followed by surgical exploration when indicated and histopathological confirmation.

## Introduction

Hydatid disease is a parasitic infection caused by the cestode Echinococcus granulosus. It is highly prevalent in regions of the Middle East, sub-Saharan Africa, and South America [1,2]. Infection is transmitted through accidental ingestion of parasite eggs, typically from contaminated food or water sources or via direct contact with infected animals. After ingestion, eggs hatch into oncospheres that penetrate the intestinal wall and enter the portal circulation, where they are trapped in the hepatic sinusoids and develop into hepatic hydatid cysts. In certain cases, they bypass hepatic trapping and reach the pulmonary circulation, or more rarely, the systemic circulation, allowing direct seeding of other organs such as the pancreas, as observed in this case. For this reason, Primary pancreatic hydatid involvement in humans is rare, occurring in less than 1% of cases [1]. Secondary peritoneal dissemination of the pancreatic cyst may follow, caused by rupture of the mother cyst and spread to the peritoneum, although extremely rare. Our case is the first case reported in the literature with this specific presentation. Clinicians may sometimes fail to correctly identify primary pancreatic hydatid involvement, especially in cases with secondary dissemination, as mass effect symptoms may mask the original pancreatic pathology.

## Case presentation

A previously healthy 29-year-old male came to the emergency department in our hospital on April 4, 2024, with severe lower abdominal pain persisting for two days. He was referred to us by a private clinic due to abnormal ultrasound findings indicative of suspected dilation of the right renal pelvic calyceal system. The patient reported burning micturition and increased urinary frequency accompanying the abdominal pain, which initially suggested a possible genitourinary etiology. A review of the systems was otherwise unremarkable. General physical examination was normal. The abdomen revealed visible distention, generalized rebound tenderness, rigidity, and guarding upon palpation. Bowel sounds were audible on auscultation with no additional sounds. These findings on examination were consistent with peritonitis and raised concern for an underlying acute intra-abdominal inflammatory or surgical process. Laboratory findings upon admission are summarized below (Table 1). 

**Table 1:** Laboratory findings on admission No validated scoring systems or proprietary assessment tools were used in this case report.

Category	Parameter	Result	Reference Range
Inflammatory markers	White blood cell count	>14,000 /µL	4,000–11,000
	C-reactive protein (CRP)	199.8 mg/L	<5
	Procalcitonin	0.75 ng/mL	<0.1
Renal function test	Creatinine	1.36 mg/dL	0.6–1.2
Electrolytes	Sodium	135 mmol/L	135–145
	Chloride	96 mmol/L	98–106
	Anion gap	13 mmol/L	8–16
Liver function tests	Total bilirubin	1.71 mg/dL	0.3–1.2
	Alanine aminotransferase (ALT)	71 U/L	<40
	Gamma-glutamyl transferase (GGT)	80 U/L	<55
	Globulin	3.8 g/dL	2.0–3.5
Pancreatic enzymes	Lipase	45.6 U/L	0—160
Urinalysis	Protein	1+	Negative
	Ketones	2+	Negative
	Urobilinogen	1+	Negative

**Figure 1:**
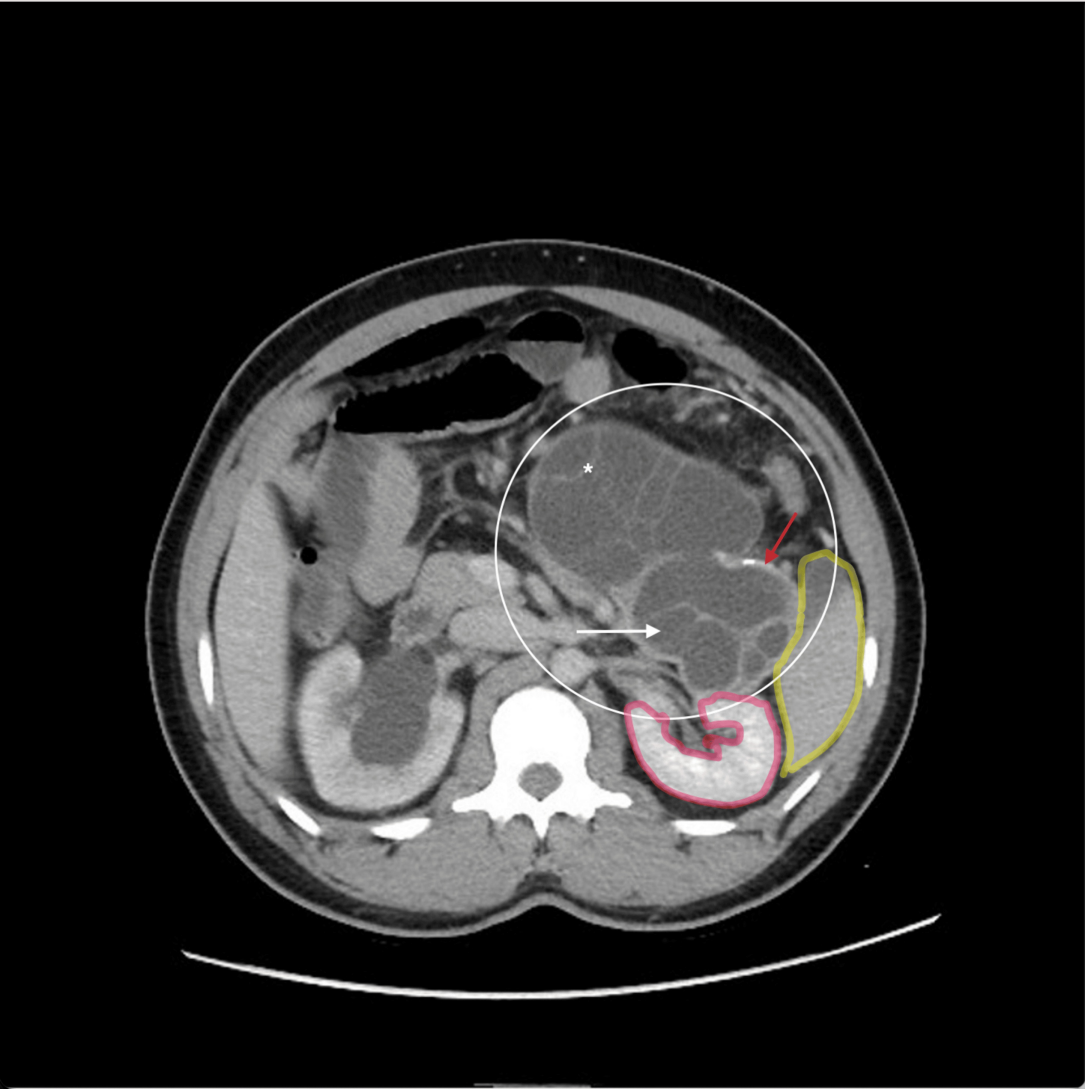
CT scan of the abdomen (axial view) The image demonstrates a dominant multiloculated cystic lesion arising from the pancreatic tail with internal separations (asterisk) and daughter cyst architecture (white arrow), consistent with World Health Organization Informal Working Group on Echinococcosis (WHO-IWGE) cystic echinococcosis (CE)2 hydatid cyst morphology. Focal peripheral calcification is noted (red arrow). Spleen (yellow highlight) and kidney (red highlight) can also be visualized.

In the presence of marked leukocytosis and significantly high CRP and procalcitonin levels, the working diagnosis at this stage favored an acute inflammatory or infectious intra-abdominal etiology. Normal lipase levels helped rule out acute pancreatitis as a primary diagnosis, and the mild elevations in bilirubin and liver enzymes were thought to be mostly reactive and nonspecific. Urinalysis findings alone were insufficient to explain the abnormal abdominal findings, prompting us to proceed with further imaging scans. A CT scan of the abdomen and pelvis with contrast done in the emergency room was evident of a large multiloculated cyst measuring 9 cm in dimension with multiple septations and focal peripheral calcification seen in the region of the pancreatic tail, which could not be clearly separated from the spleen highlighted in yellow or the kidney highlighted in red in Figure 1, suggesting extensive attachment. A cyst was noted with similar characteristics measuring about 9 cm in dimension in the region of the left flank. Multiple cysts were noted in the mid and lower abdomen, mainly on the left side, with one cyst in particular compressing the bladder, resulting in bilateral hydronephrosis. Other abdominal organs were unremarkable. Imaging findings of multiple cysts with internal separations and peripheral calcification were more consistent with hydatid disease and less supportive of a pancreatic pseudocyst, as the latter lacks calcified walls and daughter cysts. Similarly, a diagnosis of mucinous cystadenoma was excluded because the extra-pancreatic extension and involvement suggested a parasitic etiology.

**Figure 2:**
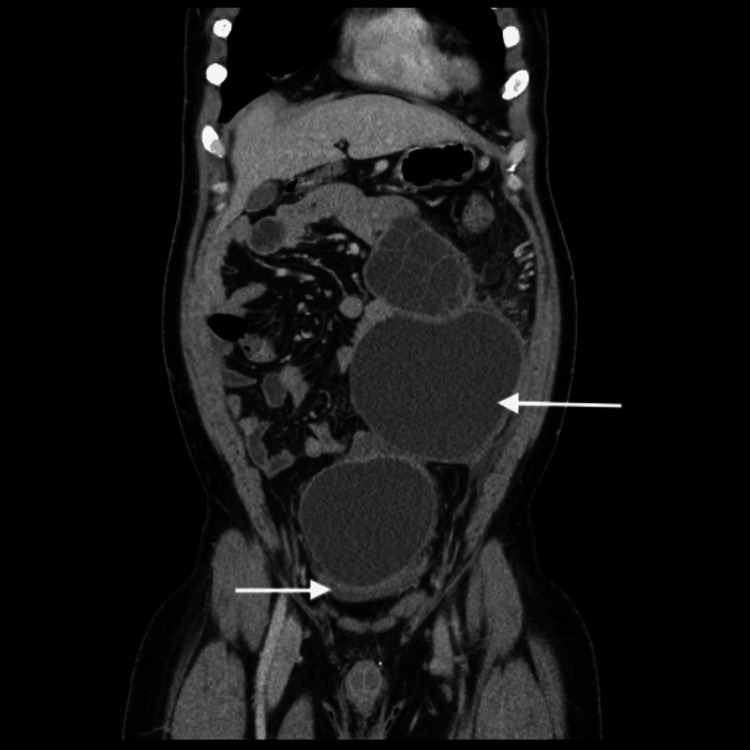
Abdominopelvic CT scan image (coronal view) Coronal CT scan image shows multiple intra-abdominal and pelvic cystic lesions consistent with secondary peritoneal dissemination. A large pelvic cyst is seen compressing the bladder (lower arrow).

Furthermore, the pancreatic cyst localized to the tail had the same characteristic features of a cystic echinococcosis (CE2) cyst in morphology according to the World Health Organization Informal Working Group on Echinococcosis (WHO-IWGE) classification, whereas the disseminated intra-abdominal cysts followed a CE1 cyst pattern with no internal septations or daughter cysts visualized, suggesting likely previous acute rupture with dissemination. These imaging findings strengthened the likelihood that this was a primary pancreatic hydatid disease complicated by rupture (Figure 2).

The clear presence of multiple cystic lesions on imaging called for involvement and referral to the general surgery team to further investigate their etiology. In view of the resulting hydronephrosis caused by cystic compression of the bladder, the general surgery team consulted the urology team and was instructed to monitor creatinine levels and to initiate continuous monitoring for any deterioration or decline in kidney function, which would necessitate the placement of a ureteral stent. The case was then admitted under general surgery for additional workup. Retrospectively, the history was revised with our patient confirming his past and prolonged contact with a guard dog back in his home country, prompting us to send a venous blood sample to assess Echinococcus immunoglobulin (Ig)G serum levels due to his suspicious demographic and direct exposure. The general surgery team then took the patient for aspiration and drainage of one large cystic lesion within the left lower abdomen due to the cyst’s considerably large size and to study the etiology of the aspirated peritoneal fluid. 

Our patient was concomitantly started on pharmacological treatment with 400 mg chewable albendazole tablets twice daily for two weeks at the same time. The puncture, aspiration, injection, and re-aspiration (PAIR) technique was done, and peritoneal fluid was aspirated under ultrasound guidance from the large cystic lesion within the left lower abdomen under local anesthesia and then sent for cultures right after which later came back negative, showing no aerobic, anaerobic, or Gram stain growth. The specimen was sent for cytological examination and revealed inflammatory and necrotic cellular debris with parasitic-like structures, and with no identified malignancy. The Echinococcus IgG venous blood sample (ELISA) had also returned positive, indicating past or current hydatid infection. A repeat CT scan was done after the procedure. The previously seen small, anteriorly located, loculated cyst in the left lumbar region is no longer visualized. A small anatomical cyst has regressed, and no other significant interval change has been detectable. Despite initial efforts with PAIR and medical therapy, the persistent large cyst burden with ongoing mass effect and the multiplicity of disseminated intra-abdominal cysts rendered surgery to be the most appropriate and time-sensitive definitive management before completion of the full medical course of albendazole. 

Intraoperative findings showed a complex hydatid cyst at the tail of the pancreas adherent to the adjacent left kidney, adrenal gland, spleen, and mesentery. The extensive adhesions and multi-organ involvement both explained the prior failure of non-invasive management with PAIR and pharmacological therapy alone. Intraoperative findings confirmed the presence of a large 9 cm dominant cyst arising primarily from the pancreatic tail with fixed adhesions to other adjacent structures along with many daughter cysts found in the abdominal cavity consistent with gross features of the hydatid cyst thick and white laminated wall and detachable inner germinal membrane most probably spread from the acute preceding dominant cyst rupture which helped explain the patient's presentation of acute abdomen and pelvic mass effect. The size, complexity, and strong attachment of the mentioned cyst strongly supported a primary pancreatic involvement with secondary peritoneal dissemination of daughter cysts rather than primary intra-peritoneal or hepatic disseminated disease. Additionally, the absence of any identified hepatic cysts on imaging and during exploration strengthened our definitive diagnosis of primary pancreatic disease with secondary dissemination. Due to the multiorgan involvement and dense adhesions to the kidney, adrenal gland, and spleen, we decided against an R0 distal pancreatectomy and instead dissected the mother cyst on the tail of the pancreas and cauterized then sutured of the raw area of the pancreas followed by excision of the maximum number of secondary cysts and injection of a scolicidal agent to prevent any future spontaneous or iatrogenic secondary rupture after surgery. A distal pancreatectomy was not appropriate in this case, as the risks of pancreatic fistula or another surgical rupture outweighed the benefits. Cysts excised from omentum and peritoneum were sent to histopathology for assessment and confirmation of hydatidosis (Figure 2), and the abdomen was closed in layers with two abdominal drains inserted in place. 

**Figure 3:**
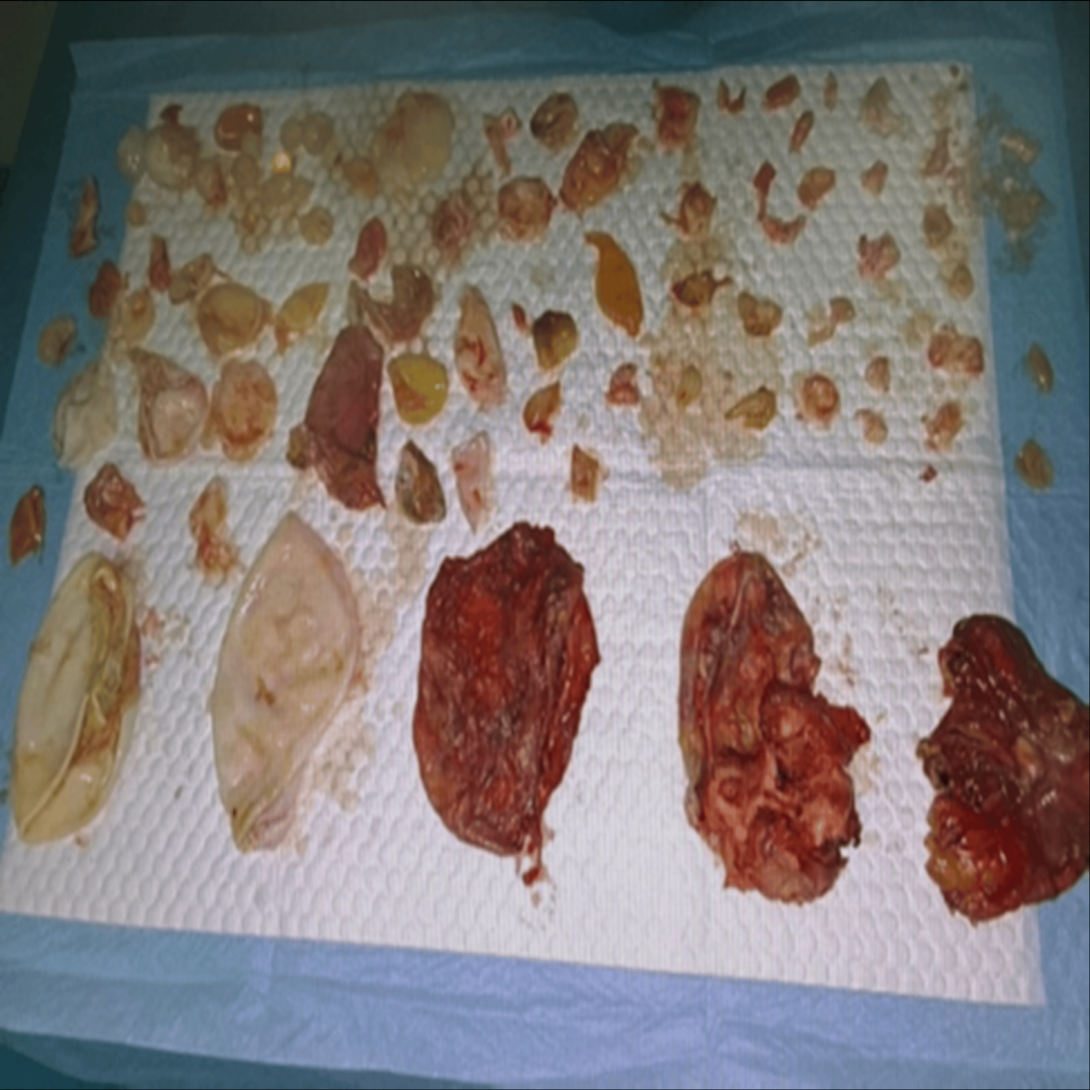
Excised secondary peritoneal and omental hydatid cysts following rupture of primary pancreatic cyst

**Figure 4:**
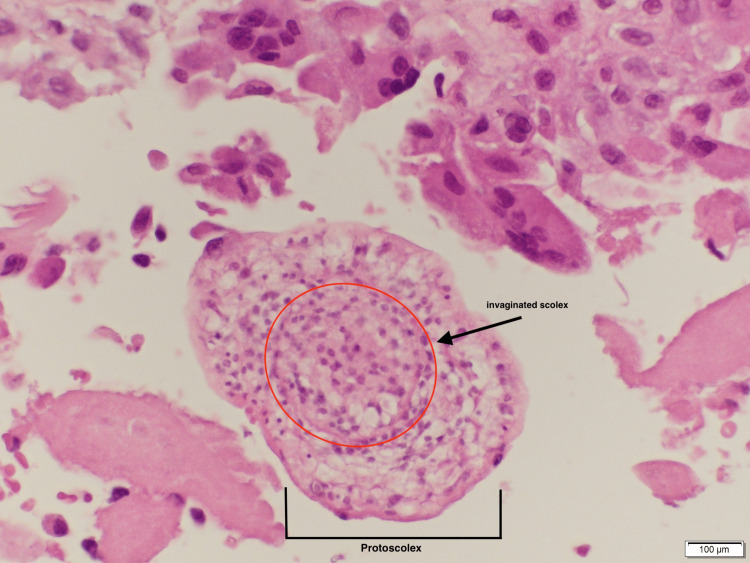
Histopathological section image confirming Echinococcosis infection Histopathology image shows a protoscolex with an invaginated scolex (circled) consistent with Echinococcosis infection (H&E stain). Scale bar=100μm.

Histopathological analysis demonstrated identifiable proctoscolices (Figure 3) and fragments of acellular laminated membrane surrounded by an outer fibrotic layer with granulation tissue (Figure 4), findings that are pathognomic for Echinococcosis infection. 

**Figure 5:**
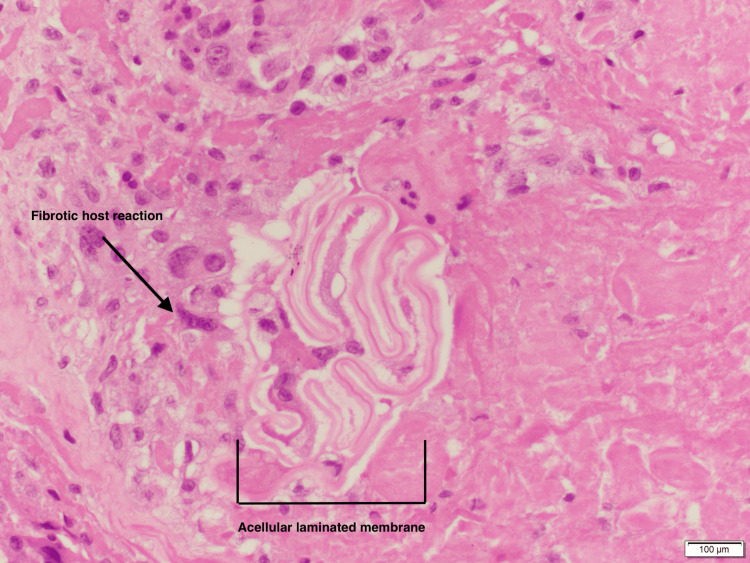
Histopathological section showing a Hydatid cyst The image demonstrates an acellular laminated membrane with surrounding fibrotic host reaction (arrow), consistent with a hydatid cyst (H&E stain). Scale bar = 100 μm.

Together with intraoperative visualization of daughter cysts and thick laminated walls, and features correlating with a multiloculated cystic appearance with internal separations observed on prior C.T. imaging, histopathology definitively confirmed the diagnosis of hydatid disease and supported our decision in excluding cystic neoplasms or pseudocysts in the early stages of investigations. Postoperatively, the patient was resumed on albendazole therapy. The immediate postoperative course showed persistent drainage and elevated inflammatory markers reflecting the extent of involvement and ongoing inflammation visualized during surgery. The patient was also started on broad-spectrum antibiotics and had a prolonged post-operative state. The patient improved over the course of the next two weeks in terms of stabilization of vital signs and a decrease in inflammatory markers, with general improvement of symptoms. However, due to continued turbid exudate fluid drainage from the pelvic cavity, the decision was made to discharge him with a pelvic drain in situ and close outpatient follow-up to monitor healing progress. 

## Discussion

Primary pancreatic hydatid disease is an exceptionally rare manifestation of the echinococcosis infection accounting for less than 1% of cases mentioned in literature [1]. This case is even more uncommon, with secondary peritoneal dissemination resulting from the acute rupture of the pancreatic dominant cyst and an atypical clinical presentation. The radiologic appearance of hydatid cystic pancreatic lesions poses a diagnostic challenge, as the latter may closely resemble pseudocysts or cystic neoplasms in shape. In this case, contrast-enhanced CT demonstrated a dominant multivesicular cyst in the pancreatic tail with daughter cyst morphology (CE2 pattern), alongside additional unilocular peritoneal cysts (CE1 morphology), raising initial suspicion for disseminated hydatid disease and effectively ruling out pseudocysts or mucinous cystadenomas due to distinctive morphology for hydatid disease. Unlike malignant cystic neoplasms, where resection is the goal of treatment, hydatid disease requires a more conservative approach focused on cyst excision, especially in cases with extensive attachment to other organs, making resection very difficult and potentially damaging. Scolicidal agents must also be injected during surgery to prevent recurrence of this complication caused by secondary spontaneous or iatrogenic rupture of evacuated cysts during or after surgery, especially in not yet fully calcified cysts of CE1 and CE2 morphology. Therefore, a distal pancreatectomy would not be indicated here. Combined surgical clearance and Albendazole therapy for at least four months remains the cornerstone of management in complex disseminated hydatid disease. [2]

Patients with the disease are usually asymptomatic until complications caused by primary mass effect and compression of adjacent organs or secondary to rupture of daughter cyst [1]. Reported clinical manifestations in a case of primary pancreatic hydatid cyst include abdominal pain in the epigastric or left upper quadrant regions along with fever, vomiting, weight loss, and signs & symptoms of biliary or pancreatic duct obstruction, such as itchy skin, dark urine, and pale stool more commonly reported in cases of cysts located in the head of the pancreas [3,4]. These symptoms may be masked by the acute presentation upon rupture, which may further delay diagnosis and negatively impact prognosis. Exposure history to animal hosts like dogs and cattle or travel to endemic areas greatly helps clinicians suspect hydatid disease and consider pancreatic hydatidosis in the differential diagnosis of cystic pancreatic lesions in cases where imaging is strongly diagnostic and urgent intraoperative exploration is contraindicated in high-risk cases or not widely available, particularly in primary care centers of endemic regions. This case emphasizes the importance of considering hydatid disease in atypical pancreatic cystic lesions, particularly in endemic regions, and highlights the value of correlating imaging morphology with intraoperative and histopathologic findings.

## Conclusions

This case illustrates primary pancreatic hydatid disease with secondary peritoneal dissemination and acute urinary mass effect following probable cyst rupture. Characteristic morphology on imaging, later confirmed by intraoperative exploration and histopathological analysis allowed the distinction from other differentials like cystic neoplasms and pseudocysts facilitating guided and appropriate surgical management. Early recognition of this rare presentation is essential to limit misdiagnosis. Long-term treatment with albendazole must also be initiated as early as possible after surgery to maximize surgical outcomes and improve overall prognosis. 
